# Composition and dynamics of macroinvertebrates community in relation to physicochemical parameters of hydrogeologically connected wetlands in Abbay River basin, Ethiopia

**DOI:** 10.1371/journal.pone.0314969

**Published:** 2024-12-09

**Authors:** Getachew Fentaw, Getachew Beneberu, Ayalew Wondie, Belachew Getnet

**Affiliations:** 1 Department of Fisheries and Aquatic Science, College of Agriculture and Environmental Sciences, Bahir Dar University, Bahir Dar, Ethiopia; 2 Department of Biology, College of Science, Bahir Dar University, Bahir Dar, Ethiopia; 3 Lake Tana and other Water bodies Protection and Development Agency, Bahir Dar, Ethiopia; 4 Faculty of Social Sciences, Bahir Dar University, Bahir Dar, Ethiopia; King’s College London, UNITED KINGDOM OF GREAT BRITAIN AND NORTHERN IRELAND

## Abstract

Assessing the macroinvertebrate assemblage in relation to physicochemical parameters can provide insight into the ecological state of aquatic environments. Therefore, this study aimed to assess macroinvertebrate assemblage of hydrogeologically connected wetlands in relation to physicochemical water quality parameters. Data were collected between June 2022 and April 2023 from twelve purposively selected sampling sites following established procedures. A total of 1,211 macroinvertebrates were collected from 18 orders and 44 families. The majority (72.83%) are generally pollution-tolerant families of the order Hemiptera, Odonata, Coleoptera and Diptera. There was significant spatio-temporal variation (P < 0.05, One-way ANOVA) in total macroinvertebrate abundance and bioindices. There were more individual macroinvertebrates collected during the dry season. The CCA and correlation analysis indicated that the physicochemical parameters had an effect on the distribution and abundance of macroinvertebrates. The size of the wetlands andthe intensity of anthropogenic interventionmight also result difference in macroinvertebrate abundance across the wetlands. The higher nutrient concentrations, the low DO level, the higher abundance of tolerant taxa and the medium Shannon_Hvalue (range: 2.13 to 2.68) all indicate the wetlands’ poor ecological status. Therefore, regular water quality monitoring, identification of the macroinvertebrate at the lower taxonomic level and the development of macroinvertebratebased multimetric indices are recommended for their sustainable management.

## 1. Introduction

Wetlands are an important part of the landscape [1] that provide many ecological services and socio-economic benefits [2]. However, many are threatened, mainly by anthropogenic disturbances [2]. As per Ramsar Convention Secretariat [3] 35% of global wetlands have been lost since 1970. These disturbances have an impact on the ecosystems’ ecological integrity [4] as well as the services offered by these ecosystems. Therefore, assessment and monitoring of wetlands’ ecology is relevant for their wise use and maintenance of their ecological character [5]; and it also provides information to support management decisions [6].

Anthropogenic disturbance of aquatic ecosystems has been assessed using abiotic indicators or physicochemical water quality measures [7–9]. However, physicochemical water quality measures provide snapshots of the condition of a water body at the time of sampling [10]. Associated monitoring is also quite costly as it requires regular data collection over long periods of time, sophisticated laboratory equipment, and highly skilled personnel [10, 11]. Furthermore, physicochemical water quality parameters are unable to give reliable early warning signals on resource condition to aquatic resource managers [8]. Thus, biotic indicators are preferable, as they provide a direct measure of ecological integrity by integrating various stressors [12–14]. In addition, they are very appropriate for developing countries like Ethiopia, where the allocation of budget and materials are inadequate for collecting continuous time series physicochemical data [7].

The use of bioindicators for assessment and monitoring of freshwater ecosystems in Ethiopia, however, is still in its infancy stage [6, 7, 15]. It is applied to assess the ecological status of only few wetlands, and streams and rivers, even though the country has vast wetland resources in 12 river basins [16]. Most studies that used macroinvertebrates as indicators (e.g., [17–23]) were cross-sectional surveys that do not take into account the temporal variability of macroinvertebrate abundance. According to Muralidharan et al. [24], the samples of the macroinvertebrate community that are taken seasonally can portray the effects of pollutant sources better than the cross-sectional sampling. According to Rethinam Subramanian et al. [25], taking the macroinvertebrates at a time (either wet and dry season) could not clearly provide the dynamics of the macroinvertebrates and the real ecological conditions of aquatic ecosystem. It would only show the macroinvertebrates assemblage and the ecological status of a time when sampling is taken. Other authors (e.g., [12, 19, 26, 27]) have reported that assessment of the structure and composition of macroinvertebrates in relation to physicochemical parameters gives important clues on the ecological status of wetlands. Sims et al. [28] argued that integrating biological and physicochemical parameters are preferable to using either biological or physicochemical parameters to display the overall ecological conditions of freshwater ecosystem and identify the point and non-point sources of pollutants. Taking this into account, this study analyzes the macroinvertebrate composition across hydrogeologically connected wetlands in relation to physicochemical water quality parameters. The findings of this study will be fundamental in providing valuable information about the ecological status of the study wetlands and hence, it will contribute to policy-making for the protection and sustainable management of wetlands in the study area. It will also be used as a framework for other similar studies particularly for those who will conduct comparative (spatio-temporal) assessment on the relationship between macroinvertebrates and physichochemical parameters of wetlands and other freshwater ecosystems.

## 2. Materials and methods

### 2.1. Description of study wetlands

**Fig 1:**
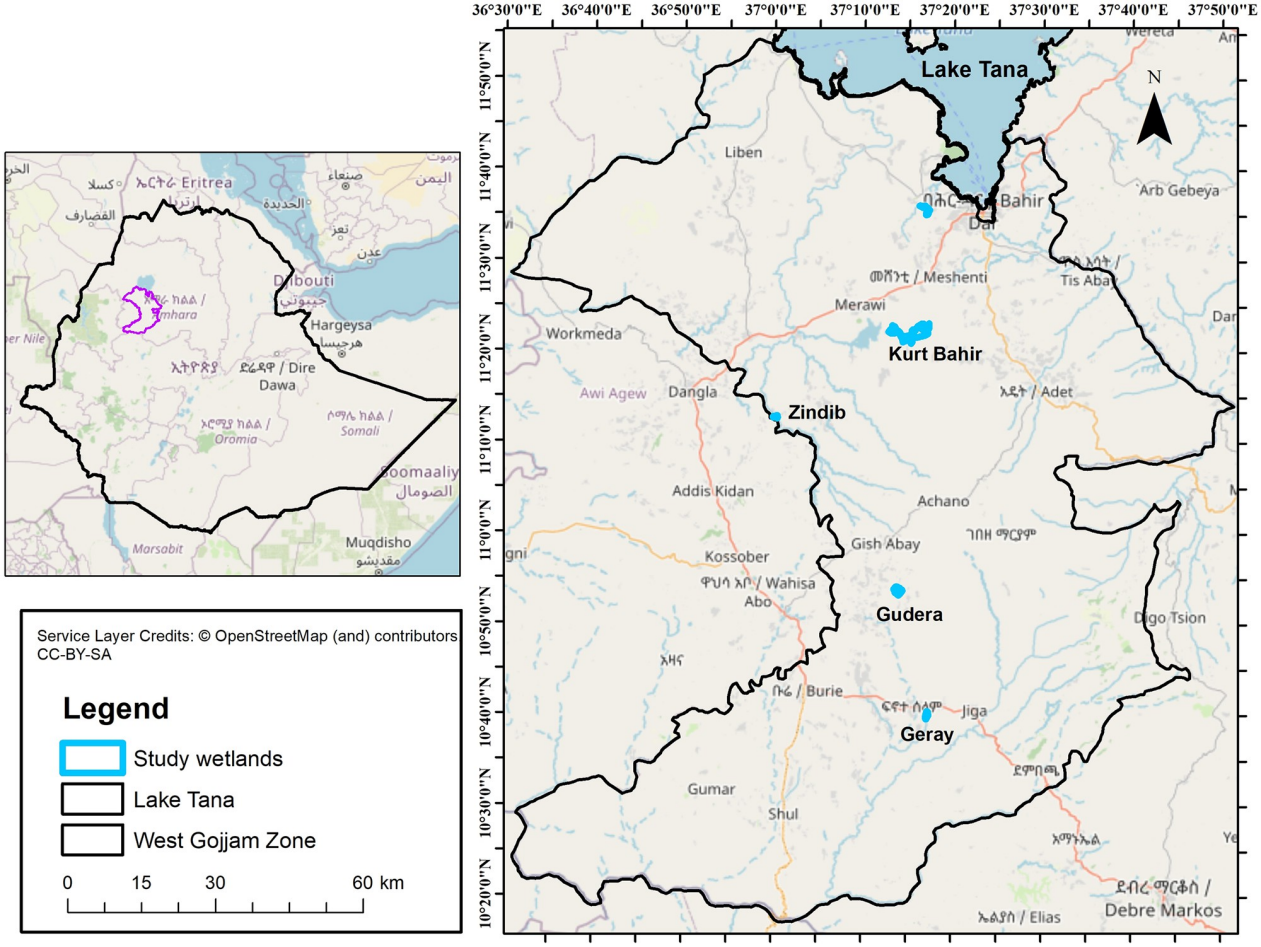
Location map of west Gojjam zone showing the six study wetlands(Attribution: Background data source is ©OpenStreetMap 2024-10-31).

The study was carried out in six wetlands; Geray, Gudera, Zindib, Kurt Bahir, Infranz and Wonjeta. They are located in Abbay River basin within west Gojjam administrative zone of Amhara region, Ethiopia (Fig 1; Table 1). They share and are located along a rocky-bush land landscape feature, possibly suggest hydrogeological connection. The local communities have also an indigenous knowledge on the hydrological connectedness between Gudera-and-Geray and Kurt Bahir-and-Infranz wetlands (personal communications). The studied wetlands do not have surface water connection, making them geographically isolated. However, geographical isolation does not mean hydrological, biogeochemical, or biological isolation from other landscape elements [29, 30]. Therefore, they might be at least hydraulically connected to the water table aquifer via groundwater.

**Table 1:** Location of studied wetlands and description of sampling sites.

Wetland	Area (ha)	Woreda (District)	Bordering Kebele (Villages)	Coordinate		Elevation (m)	Description of sampling site
				North	East		
**Geray**	10	Jabitehnan and Finoteselam	Arbaitu-Ensesa (Jabitehnanworeda) and Shembekuma-Yidafas (Finoteselamworeda)	10°40’22.5"	37°17’16.0"	1789	The site is close to settlement (Addis-amba village). It is well vegetated but disturbed by water abstraction for domestic use, cloth washing and animal watering.
				10°39’30.2"	37°17’7.9"	1802	It is the reservoir highly disturbed by swimming, bathing, fishing, and water abstraction for irrigation.
**Gudera**	140	Sekela	Asewa T/haimanot and Zegeza-tsengafakebeles	10°53’7.9"	37°14’1.7"	2352	The site is in Asewa T/haimanot kebele. Recession agriculture is the main threat. Free grazing and livestock watering are also common.
				10°53’20.1"	37°14’12.1"	2344	It is in Zegeza-tsengafa Kebele impacted by free grazing, livestock watering and dumping of animal waste.
**Zindib**	28.55	North Mecha	Nada Maryamkebele, Dil-Betigilvillages	11°12’40.6"	37°0’9.8"	2059	The site is the head (source) of the wetland and characterized by free livestock grazing.
				11°12’44.6"	37°0’0.6"	2057	It is the site where water drains into GilgelAbay River during the rainy seasons. Free grazing and farming are the main threats.
**Kurt Bahir**	764	North Mecha	Kurt-Bahir, Midre-Genet, Tatek-Geberie, and Enashenifalen villages	11°21’47.7"	37°16’38.8"	2052	The site is in Tatek-Gebere village where free grazing, fodder collection, livestock watering, and farming are common.
				11°22’7.6"	37°12’59.8"	2049	It is in Kurt Bahir kebele; cloth washing and bathing during the rainy season (when water drains into Koga dam). Relatively no free grazing, feed their cattle through cut-and-carry system.
**Infranz**	25,750	Bahir Dar zuria	Infranzkebele	11°35’43.8"	37°16’33.6"	1831	The site is one of the springs (locally called Lomi-minch) where water is abstracted for supplying drinking water for Bahir Dar city and for the locals. It has a good coverage of macrophytes but highly impacted by excavation.
				11°37’15.5"	37°17’22.1"	1811	This site is the Infranz river mouth, threatened by cloth washing, livestock watering, farming, etc.
**Wonjeta**	300	Bahir Dar Zuria	Wonjetakebele	11°41’34.5"	37°16’44.7"	1800	The site is locally named as ‘Eslam-minch’ with relatively good coverage of macrophytes, dominated by *Cyperus*spp. and less impacted by human activities.
				11°41’35.0"	37°16’45.2"	1797	The site (‘Tekuma’) is covered by *Azolla* and threatened by bathing, cloth washing, water abstraction for domestic and irrigation use, livestock watering and free grazing are common.

**Source:** Fentaw et al.(2024)

### 2.2. Sample site selection and data collection

A total of 12 sampling stations were established across the six studied wetlands; two stations from each wetland (Table 1). They were purposively selected from heavily and less disturbed sites using human disturbance parameters (intensity of hydrological modifications, habitat alteration, and land use practices)as criteria [31, 32]. The macroinvertebrates data was collected during wet (July to September, 2022) and dry (January to March, 2023) seasons. The results of our previous study [33] on the physicochemical features of the study wetlands have been used as a secondary data.

### 2.3. Macroinvertebrate sampling and identification

Multihabitat sampling approach was employed to collect the highest possible diversity of macroinvertebrates [34]. Sampling was carried out following protocols for sampling macroinvertebrates in freshwater wetlands [35]. A travelling kick and sweep method was employed [17] at each sampling stations using a handheld D-framed kick net during the wet and dry seasons. Equal sampling effort (30 minutes) was allotted to cover the different micro-habitats [36, 37]. The bottom sediment was disturbed during sampling and the collected macroinvertebrates were pooled into a single composite sample from each wetland.

In the field, the collected material was sieved through 500 μm and 250 μm mesh sieves and emptied into a rectangular tray; then a hand picking method using forceps was employed to sort macroinvertebrates [37, 38]. All samples were preserved in labelled vials containing 70% ethanol and transported to the laboratory of Bahir Dar Fishery and Other Aquatic Life Research Centre for further analysis. Identification was carried out to the family level using a stereomicroscope (x10 magnification) and different identification keys (e.g., [27, 39, 40]), and finally individual families were counted.

### 2.4. Ethical approval

The research proposal was presented to the department of Fisheries and Aquatic Sciences, School of Fisheries and Wildlife, Bahir Dar University. Then the proposal was evaluated and approved by the departmental graduate council. After this, the school research ethics review committee approved the proposal and give written permission for fieldwork and collection of macroinvertebrates (ethics clearance reference number: FASc-24/14/2016).

### 2.5. Data analysis

Diversity indices were computed to provide information on the structure of macroinvertebrate assemblages of the study wetlands using PAST4.13 software. Spatio-temporal variations in the measured bio indices across the study wetlands and seasons were assessed using one-way ANOVA; after normality of the data were checked using Shapiro-Wilk test (p > 0.05) using SPSS version 20. Canonical correspondence analysis (CCA) was performed using CANOCO 4.5 to investigate the relationship of macroinvertebrates abundance with physicochemical water quality parameters on ordination axes. The correlation between physicochemical parameters and macroinvertebrate indices was also analyzed (Pearson’s correlation). The macroinvertebrate data was transformed (log square) to normalize the distribution and homogenize the variances.

## 3. Results

### 3.1. Spatio-temporal variation in macroinvertebrate abundance and diversity

#### Spatial variation in macroinvertebrate abundance and diversity

A total of 1,211 macroinvertebrates belonging to 18 orders and 44 families were collected from all the six studied wetlands (Table 2) during the wet and dry seasons. From the total number of individuals, the highest number of individuals (282, 222, and 205) was recorded in Gudera, Infranz, and Kurt Bahir wetlands, respectively. The order Hemiptera was the most dominant (475individuals), abundant (39.2%) and diverse (comprised 7 families); followed by Odonata (295), Coleoptera (187), and Diptera (112 individuals). Notonectidae and Corixidae were the most dominant families (RA: 20.23% and 13.29%, respectively) among the order Hemiptera that were identified in all the studied wetlands. Coenagrionidae and Gomphidae were the most dominant families within the order Odonata, which constituted 8.51%, 8.42%, respectively, whereas Dytiscidae was the dominant family within the order Coleoptera (represented 9.66%) and Chironomidae was the most dominant family within the order Diptera (represented 9.0%).

**Table 2:** Number of families and individuals found within each main taxonomic group in the six study wetlands.

No	Order	Geray		Gudera		Zindib		K Bahir		Infranz		Wonjeta		Total		RA
		# family	individual	# family	Individual	# family	Individual	# family	Individual	# Family	individual	# family	Individual	# family	Individual	
1	Coleoptera	2	25	2	24	3	33	5	35	4	34	4	36	6	187	15.44
2	Diptera	2	6	3	35	1	1	1	36	1	2	1	32	4	112	9.25
3	Ephemeroptera	0	0	1	1	0	0	1	5	2	6	1	2	3	14	1.16
4	Hemiptera	6	71	6	152	4	77	4	79	5	37	4	59	7	475	39.22
5	Lepidoptera	1	1	0	0	0	0	0	0	0	0	0	0	1	1	0.08
6	Odonata	5	44	5	20	5	42	7	34	7	128	4	27	7	295	24.36
7	Plecoptera	0	0	0	0	0	0	1	5	0	0	0	0	1	5	0.41
8	Trichoptera	0	0	0	0	0	0	1	2	2	2	0	0	3	4	0.33
9	Decapoda	0	0	0	0	1	9	1	2	1	1	0	0	2	12	0.99
10	Araneae	0	0	0	0	0	0	0	0	0	0	1	1	1	1	0.08
11	Opisthopora	0	0	0	0	0	0	0	0	1	1	1	1	1	2	0.17
12	Hirudinida	1	1	1	3	0	0	0	0	0	0	1	1	1	5	0.41
13	Basommatophora	1	1	1	2	0	0	1	4	1	3	1	10	1	20	1.65
14	Hygrophila	1	2	1	3	2	7	1	3	2	7	2	10	2	32	2.64
15	Neotaenioglossa	0	0	0	0	0	0	0	0	1	1	0	0	1	1	0.08
16	Sphaeriida	0	0	1	34	0	0	0	0	0	0	1	3	1	37	3.06
17	Venerida	0	0	1	6	0	0	0	0	0	0	0	0	1	6	0.50
18	Ascaridida	0	0	1	2	0	0	0	0	0	0	0	0	1	2	0.17
**Total**			**151**		**282**		**169**		**205**		**222**		**182**	**44**	**1211**	

The summary of macroinvertebrate metrics across wetlands presented in Table 3 revealed that the value of Shannon-H index ranged from 2.132 to 2.68even though it was not found to be statistically significant. As per Cavalcant and Larrwazbal cited in Atsbha et al [41], the macroinvertebrates diversity was medium(between 2.0 and 3.0) in all six wetlands. Among the diversity indices, only evenness showed statistically significant difference (*F* = 3.493, *P* = 0.08) with lowest and highest values recorded in Gudera and Wonjeta wetlands, respectively.

**Table 3:** Spatial variation in macroinvertabrates taxa, abundance and diversity indices in six study wetlands.

	Geray	Gudera	Zindib	K Bahir	Infranz	Wonjeta	F-value
Taxa_S	19	23	16	23	27	21	.264
Individuals	151	282	169	205	222	182	.237
Shannon_H	2.268	2.132	2.322	2.443	2.68	2.659	.594
Evenness_e^H/S	0.5086	0.3667	0.6374	0.5004	0.54	0.6804	3.493*

#### Temporal variation in macroinvertebrate community

The summary of the macroinvertebrate communities across seasons are presented in Table 4. As presented in the table, the higher total number of individual macroinvertebrates was recorded during the dry season (852) compared to thewet season (359 individuals) from the two sampling stations. Order Hemiptera was most dominant in both dry and wet seasons, followed by Odonata and Coleoptera. These groups represented 39.6%, 24.7% and 13.7% of total number of individuals counted during the dry season, respectively, and 38.4%, 19.5% and 23.7% of total individuals recorded during the wet season, respectively.

**Table 4:** Family richness and abundance of macroinvertebrates across seasons.

No	Order	Wet season		RA	Dry season		RA
		# of family	# indv		# of family	# indv	
1.	Coleoptera	3	70	19.50	5	117	13.73
2.	Diptera	2	32	8.91	3	80	9.39
3.	Ephemeroptera	1	3	0.84	2	11	1.29
4.	Hemiptera	5	138	38.44	7	337	39.55
5.	Lepidoptera	0	0	0.00	1	1	0.12
6.	Odonata	4	85	23.68	7	210	24.65
7.	Plecoptera	0	0	0.00	1	5	0.59
8.	Trichoptera	2	3	0.84	1	1	0.12
9.	Decapoda	2	10	2.79	1	2	0.23
10.	Araneae	0	0	0.00	1	1	0.12
11.	Opisthopora	1	1	0.28	1	1	0.12
12.	Hirudinida	1	1	0.28	1	4	0.47
13.	Basommatophora	1	1	0.28	1	19	2.23
14.	Hygrophila	2	15	4.18	2	17	2.00
15.	Neotaenioglossa	0	0	0.00	1	1	0.12
16.	Sphaeriida	0	0	0.00	1	37	4.34
17.	Venerida	0	0	0.00	1	6	0.70
18.	Ascaridida	0	0	0.00	1	2	0.23
	**Total**		**359**			**852**	

The temporal variation of bioindices (Taxa richness, Shannon-H and Evenness) of macroinvertebrates presented in Table 5 also revealed the variation in terms of seasons. As presented in the table, the scores of diversity indices were higher during the dry season than the corresponding figures of the wet season. The differences of these bioindices across the two seasons were found to be statistically significant (p<0.05) except Evenness as confirmed by analysis of mean variance.

**Table 5:** Temporal variation in macroinvertebrate taxa, abundance and diversity indices in the six studied wetlands.

Seasons	Taxa_S	Individuals	Shannon_H	Evenness_e^H/S
Wet	24	359	2.439	0.4776
Dry	38	852	2.766	0.4183
F-Value	34.087***	16.836**	14.604**	0.030

### 3.2. Relationships of macroinvertebrates with water quality parameters

**Fig 2:**
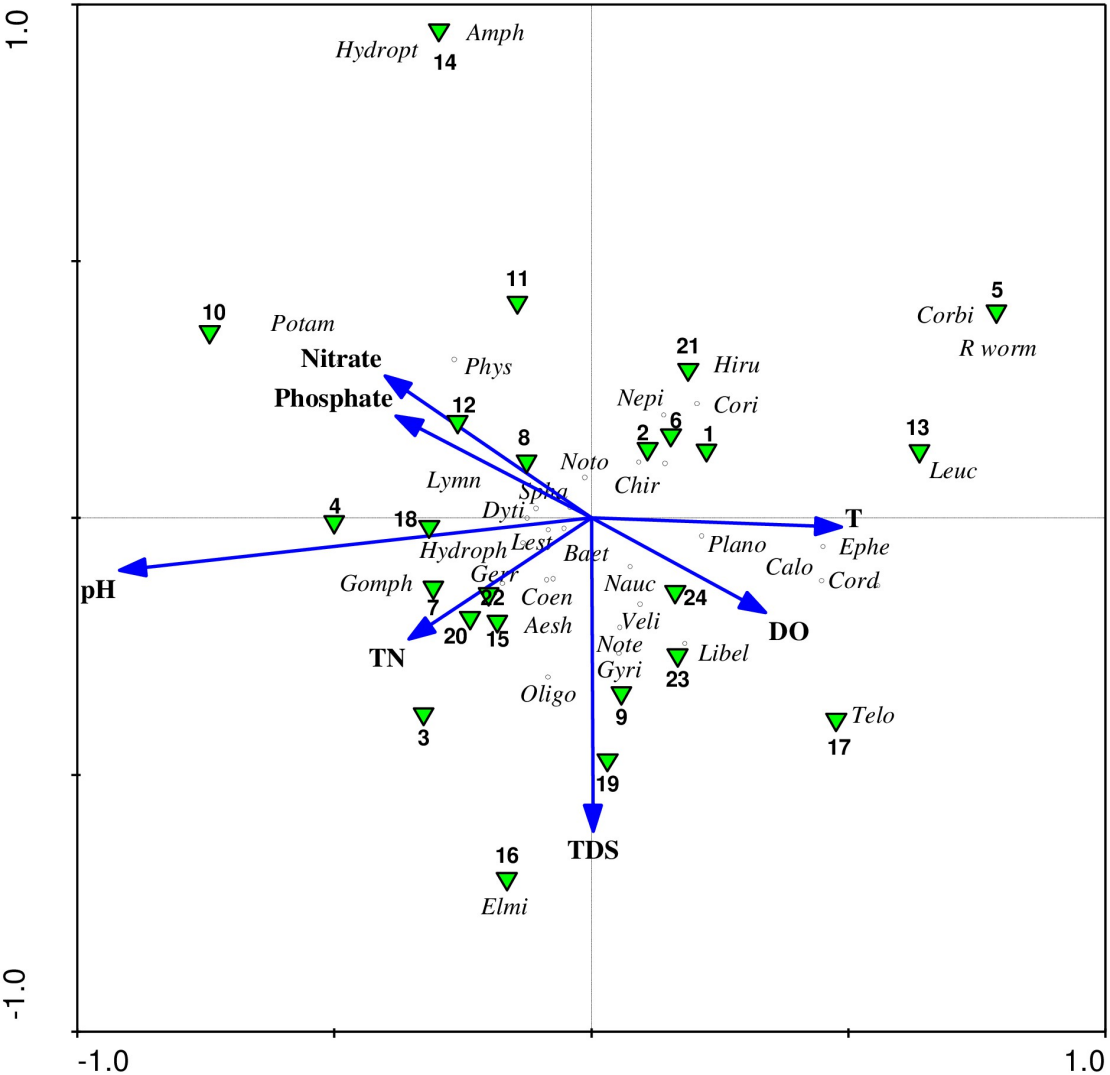
Tri-plot of first two axes of CCA for macroinvertebrates, physicochemical parameters and sampling sites. (Abbreviations: Aesh.–Aeshnidae, Amph.–Amphipoda, Baet.—Baetidae, Calo.–Calopterygidae, Chir.–Chironomidae, Coen.–Coenagrionidae,Cord.–Corduliidae, Corbi.–Corbiculidae, Cori.–Corixidae, Dyti.–Dytiscidae, Elmi.–Elmidae, Ephe.–Ephemerelidae,Gerr.–Gerridae, Gomph.–Gomphidae, Gyri.–Gyrinidae, Hiru.–Hirudinea, Hydroph.–Hydrophilidae, Hydropt.–Hydropsychidae, Lest.–Lestidae, Leuc.–Leuctridae, Libel.–Libellulidae, Lymn.–Lymnaeidae, Nauc.–Naucoridae, Nepi.–Nepidae, Note.–Noteridae, Noto.–Notonectidae, Oligo.–Oligochaeta, Phys.–Physidae, Plano.–Planorbidae, Pota.–Potamonautidae, Rworm.–Round worm, Spha.–Sphaeriidae, Telo.–Teloganodidae, Veli.–Veliidae).

The relationship between macroinvertebrate taxa distribution and physicochemical variables summarized in the CCA model explains 79.7% of the variation (Table 6; Fig 2). The first and second axes explained 47.2% of variance of species-environment relation. The first axis, which explained 24.7% of the variance, was positively correlated with T, DO and TDS but negatively correlated with pH, PO_4_^-3^ and TKN. The pH showed the strongest but negative (r = -0.85) correlation with this axis. Axis two, which explained18.7% of the variance, was positively correlated with PO_4_^-3^ and NO^-^_3_; while other variables were correlated negatively. The correlation with TDS was strong but negative (r = -0.52). The CCA also revealed that the pH, DO, TDS and PO_4_^-3^were the most important variables strongly influencing macroinvertebrate distribution.

**Table 6:** Correlation of physicochemical variables with the axes of canonical correspondence analysis (CCA).

Environmental variables	Axis 1	Axis 2	Axis 3	Axis 4
Eigenvalues:	0.247	0.187	0.168	0.131
Cumulative percentage variance of species-environment relation:	26.9	47.2	65.5	79.7
Temperature	0.4514	-0.0151	0.0082	0.107
pH	**-0.8516**	-0.0871	-0.0608	-0.2579
DO	0.3146	-0.158	**0.6791**	-0.3834
TDS	0.0031	**-0.5233**	-0.4739	0.399
PO_4_^-3^	-0.3527	0.1702	**0.6793**	0.2281
NO^-^_3_	-0.3725	0.2374	-0.061	0.4896
TN	-0.3297	-0.2025	-0.1542	-0.2062

There was also significant correlation (Pearson correlation, p<0.05) between some of the measured physicochemical parameters and the macroinvertebrates identified. There was significant negative correlation between pH and Corixidae, Corduliidae, Chironomidae, Corbiculidae and Ascarididae. Similarly, DO was negatively correlated with Hydroptilidae and Amphipoda, whereas; temperature, phosphate and TKN were significantly and positively correlated with Ephemerellidae, Potamonautidae and Elmidae, respectively. The analysis on correlation between physicochemical parameters and macroinvertebrate indices (S1 Table) also revealed a significant correlation between bioindices and some of the physicochemical parameters.

## 4. Discussion

### 4.1. Macroinvertebrate composition, abundance and diversity

Our results showed that the total number of macroinvertebrates recorded in the study seasons from the studied wetlands (1,211) was less than the corresponding figures reported in previous studies (e.g., [17, 37, 42–44]). Hemiptera were the dominant taxa that contributed the largest number of the total macroinvertebrates, followed by Odonata, Coleoptera, and Diptera. Most families in these orders are generally pollution tolerant and their dominance was also reported from other wetlands in the country (e.g., [17–19, 43–46]) in different degree.

The members of the EPT taxa (Ephemeroptera, Plecoptera and Trichoptera) are generally pollution-sensitive taxa encountered in good water quality, especially at sites with a relatively high DO concentration [14, 23, 37, 43, 47]. Their species composition and diversity decreases with increasing disturbances [12, 48], except some groups (e.g., Baetidae, Caenidae) which are tolerant for disturbances. In the present study, these pollution-sensitive taxa were represented by a relatively low number of individuals (23, 1.90%). However, about 882 (72.83%) of identified individuals belong to pollution-tolerant taxa (Hemiptera, Odonata and Diptera). This might indicate the existence of organic pollution, and thus, water quality impairment and poor ecological status of the studied wetlands. Other studies (e.g., [20, 23, 37, 49, 50]) in the country also revealed a smaller proportion of sensitive species and a higher percentage of pollution-tolerant taxa in heavily disturbed sites.

Notonectidae, Corixidae, Dytiscidae, Chironomidae, Coenagrionidae and Gomphidae were the most frequently occurring and dominant families. According to Acharyya and Mitsch [51] a dominating number of these macroinvertebrates in aquatic ecosystems often indicate high pollution load, anoxic and over enriched conditions; because of their high tolerant nature [40, 47, 52]. The dominance of these pollution tolerant species in different degree is also reported from different aquatic ecosystems in the country (e.g., [17–20, 36, 43, 44, 50]). The higher abundance of these taxa, indicates that aquatic ecosystems in the country in general and particularly the study wetlands are ecologically degraded. This is associated primarily with habitat alteration, land use change and hydrological modification [6].

### 4.2. Spatio-temporal variation in macroinvertebratecomposition and abundance

#### Spatial variation in macroinvertebrate composition and abundance

There were differences in the family richness and abundance of macroinvertebrate taxa among the wetlands. The difference in their size and intensity of anthropogenic intervention might contribute to the variation. The relatively higher macroinvertebrate abundance was recorded in Gudera, Infranz and Kurt Bahir (Table 3) than Geray, Zindib and Wonjeta. This might be due to their larger area coverage of the former (Table 1). Wetlands with larger surface area might have larger drainage basins, which result in greater nutrient input and contribute to greater macroinvertebrate richness [53]. The diversity and abundance of macroinvertebrate is also associated with habitat type [54]. Wetlands with larger surface area support higher macroinvertebrate as a result of higher habitat heterogeneity.

According to Ngodhe et al. [55] small water bodies are experiencing rapid eutrophication due to nutrient loading, sedimentation, acidification, and the introduction of toxic contaminants as a result of runoff water, which could reduce macroinvertebrate abundance. For example, Geray is a small weir primarily established for irrigation, dominated by agricultural production, which might increase phosphorus-bound sediments in a reservoir; which might explain the lowest macroinvertebrate abundance in comparison with the other wetlands. Zindib is also a small temporary wetland often used as grazing field, particularly during the dry season, resulting in the deposition of a significant amount of cattle excrements. When it becomes inundated, the dead organic material and cattle excrements can be decomposed thereby rising the concentration of total phosphorus [37, 44]; which might result in lower taxa richness and abundance.

The highest abundance of macroinvertebrate at Gudera wetland might be associated with the higher grazing pressure and silt load (from the degraded catchments, intensive recession agricultural activity and the diversion of Zegez River during the rainy season). Several studies (e.g., [17, 20, 21, 36, 37, 44, 56]) indicated that wetlands with intermediate disturbance (slightly disturbed sites) support a higher abundance and diversity of macroinvertebrates. On the other hand, human activities associated with agriculture, overgrazing and deforestation are the main cause of water quality deterioration and loss of macrophytes; causing biodiversity decline, particularly pollution-sensitive taxa [37, 44, 57]. The relatively low average diversity value (H’ = 2.1) of Gudera shows that the lake and its wetlands are polluted, due to high anthropogenic activity [38, 55]. According to Atnafu et al. [58] an excessive sediment load from the degraded catchment could be the cause for the decline of macroinvertebrates. Sedimentation deteriorates water quality and reduces light penetration, affecting primary producers, which in turn affects macroinvertebrates. The lowest evenness (E = 0.37) is due to the fact that the wetland is dominated by two families of Hemipterans: Corixidae (29.95%) and Notonectidae (25.53%). The wetland is also dominated by other pollution-tolerant taxa like Sphaeriidae (12.10%) and Chironomidae (11.7%), but devoid of the EPT group, except Baetidae, which is a pollution-tolerant taxon within the order Ephemeroptera.

The Infranz wetland had the highest taxonomic richness, abundance and diversity index value, as reported in aprevious study [44]. Gezie et al. [44] reported that Infranz had the highest abundance and diversity of macroinvertebrates compared to other wetlands in LakeTana area. The abundance and diversity of macroinvertebrates of the current study, however, was lower than the corresponding figures reported by Eneyew and Assefa [49]. The occurrence of ET taxa and the higher abundance of odonates, particularly Gomphidae (one of the sensitive families in the order Odonata) also indicated a better ecological status than the other studied wetlands. This might be associated with the wetland’s relatively better macrophyte coverage, particularly at the source of springs. Macrophytes promote the diversity of macroinvertebrates [18, 59, 60], as they provide shelter against the water current and fish predators, provide more food resources and serve as oviposition sites.

#### Temporal variation in macroinvertebrate composition and abundance

The diversity indices scores also varied significantly between seasons (Table 5). Our findings revealed that, diversity index scores were higher during the dry season than during the wet season. This is consistent with the findings of the study by Gebrehiwot et al. [61], who recorded higher macroinvertebrate abundance and richness during the dry season in the Gilgel Gibe catchment. The main reasons behind the higher abundance and richness of macroinvertebrates during the dry season in comparison to the wet season are: 1) according to Helson and Williams (2013) cited in Assefa and Eneyew [45], that the signature of maximum anthropogenic impacts is more easily detected during the dry period, 2) the high runoff that disturbed substrates and carried macroinvertebrates away, together with the build up of silt particles that hindered the development of primary producers, resulted in a scarcity of food for the primary consumers, and may be the cause of the lower macroinvertebrate abundance during the rainy season in comparison with the dry season. For example, according to Priawandiputra et al. [62], the higher abundance of molluscs during the dry season could be related to their high rates of biological activity, while during the rainy season molluscs are flushed out.

### 4.3. Relation between macroinvertebrate abundance and physicochemical parameters

Each aquatic organism has particular requirements with respect to the biological, chemical, and physical conditions of its habitat. In the present study, the measured physicochemical water quality parameters had correlation with many macroinvertebrates and influenced their distribution. This is consistent with previous studies (e.g., [18, 20, 44, 63]), which reported that physicochemical parameters are responsible for the diversity, richness and distribution of macroinvertebrates. The analysis of CCA revealed that pH, EC, TDS and DO were the most influential variables explaining the variation in macroinvertebrate assemblage patterns.

The macroinvertebrate families Potamonautidae, Physidae and Lymnaeidae were positively correlated with NO^-^_3_ and PO_4_^-3^ and restricted to sites (10, 11 and 12) in Wonjeta wetland where these nutrients were high. They are pollution-tolerant families of the order Decapoda and Gastropoda, respectively. Gerber and Gabriel [40] and Bouchard [39] reported that these macroinvertebrate families are generally pollution tolerant. On the other hand, macroinvertebrate families such as Libellulidae, Corduliidae, Calopterygidae and Teloganodidae, exhibited a positive correlation with DO but inversely related to NO^-^_3_ and PO_4_^-3^. Kabore et al. [64] concluded that these are pollution-sensitive taxa within the order Odonata and Ephemeroptera, preferring less polluted habitats characterized by relatively high DO concentrations. In fact, in our study they were restricted to sites with high DO.

Opisthoporans were detected in Wonjeta, where the lowest average DO concentration (1.70 mg/L) was recorded, indicating its high tolerance to lower DO. Their tolerance to nutrient rich water bodies and low DO level was reported in several studies (e.g., [61, 65–67]) and can be used as bioindicators of poor water quality. The results from CCA also highlighted that Physidae and Potamonautidae were found in sites 10, 11 and 12, where phosphate (1.8mg/L) and nitrate (2.73 mg/L) concentrations were high. Gouissi et al. [68] also reported that pollution-tolerant families (e.g. Physidae) were more abundant at sites where there was high phosphate and nitrate concentration.

There was significant correlation (p<0.05) between some macroinvertebrate metrics and the water quality variables across the studied wetlands (S1a Table). Phosphatewas negatively correlated with Taxa_S and nitrate negatively correlated with macroinvertebrate individuals (r = -0.847 and r = -0.856, respectively); however, the correlation with other parameters was not significant. There was also significant correlation between TP and macroinvertebrate individuals (r = -0.840) during the wet season (S1c Table). However, the correlation between bioindices and water quality parameters during the dry season was not significant (S1b Table).

## 5. Conclusion and recommendations

This study describes the community structure of macroinvertebrates of hydrogeologically connected wetlands in relation to physicochemical parameters, which varied between study wetlands and seasons. The measured physicochemical water quality parameters influenced the composition and abundance of macroinvertebrates. The CCA showed that pH, EC, TDS and DO were the most important variables that contributed to the variability in spatial community structure and composition of macroinvertebrates in these wetlands. The medium macroinvertebrate richness and diversity indicates an overall ecological degradation, caused by the presence of elevated levels of water pollution (higher nutrient concentrations and low DO level [33]). The water pollution in these wetlands is also evidenced by the presence of pollution-tolerant taxa (such as Chironomidae, Planorbidae, Earthworms) and the very low abundance of sensitive EPT taxa. Therefore, this study highlights the potential role of macroinvertebrate monitoring in identifying anthropogenic pollution. Regular sampling could help the adoption of integrated watershed management at catchment level. The establishment of a buffer zone and the application of appropriate land use planning are also very important for protecting and rehabilitating the studied wetlands. The authors also recommend the identification of the macroinvertebrate taxa to the lowest possible level and the development of macroinvertebrate multimetric indices (MMIs), which are important tools for freshwater monitoring and management.

## Supporting information

S1 Table Correlation between bioindices and physicochemical parameters across the study wetlands and seasons. (DOCX)

S2 Table Macroinvertebrates collected from the study wetlands across the study seasons. (DOCX)
