# Supplementary material for: Composition and dynamics of macroinvertebrates community in relation to physicochemical parameters of hydrogeologically connected wetlands in Abbay River basin, Ethiopia
Source: PLoS One. 2024 Dec 9;19(12):e0314969. doi: 10.1371/journal.pone.0314969 (PMC11627383; doi:10.1371/journal.pone.0314969)
Supplement: S1 Table — (DOCX) [file pone.0314969.s001.docx]

**SI_Table 1.** Correlation between bioindices and physicochemical parameters across the study wetlands and seasons

1. **The correlation between bioindices and physicochemical parameters across the studied wetlands**

|  | Temperature | pH | DO | EC | TDS | Phosphate | Nitrate | TP | TKN | Taxa_S | Individuals | Shannon_H | Evenness |
| --- | --- | --- | --- | --- | --- | --- | --- | --- | --- | --- | --- | --- | --- |
| Temperature | 1 | -.667 | -.180 | .756 | .751 | -.698 | .292 | -.331 | -.439 | .591 | -.045 | .329 | -.246 |
| pH |  | 1 | .402 | -.691 | -.661 | .728 | .219 | .548 | .626 | -.585 | -.333 | -.331 | .175 |
| DO |  |  | 1 | -.725 | -.727 | .270 | -.157 | .351 | .763 | .094 | .479 | -.686 | -.756 |
| EC |  |  |  | 1 | .999^**^ | -.637 | .296 | -.518 | -.748 | .455 | -.279 | .801 | .379 |
| TDS |  |  |  |  | 1 | -.615 | .329 | -.497 | -.746 | .433 | -.315 | .807 | .402 |
| Phosphate |  |  |  |  |  | 1 | .410 | .879^*^ | .139 | -.847^*^ | -.435 | -.465 | .282 |
| Nitrate |  |  |  |  |  |  | 1 | .595 | -.443 | -.396 | -.856^*^ | .142 | .425 |
| TP |  |  |  |  |  |  |  | 1 | .007 | -.801 | -.495 | -.633 | .029 |
| TKN |  |  |  |  |  |  |  |  | 1 | .096 | .470 | -.438 | -.499 |
| Taxa_S |  |  |  |  |  |  |  |  |  | 1 | .627 | .441 | -.403 |
| Individuals |  |  |  |  |  |  |  |  |  |  | 1 | -.226 | -.709 |
| Shannon_H |  |  |  |  |  |  |  |  |  |  |  | 1 | .638 |
| Evenness |  |  |  |  |  |  |  |  |  |  |  |  | 1 |

**. Correlation is significant at the 0.01 level (2-tailed); *. Correlation is significant at the 0.05 level (2-tailed).

**b. The correlation between bioindices and physicochemical parameters of the six studied wetlands during dry seasons**

|  | Temperature | pH | DO | EC | TDS | Phosphate | Nitrate | TP | TKN | Taxa_S | Individuals | Shannon_H | Evenness |
| --- | --- | --- | --- | --- | --- | --- | --- | --- | --- | --- | --- | --- | --- |
| Temperature | 1 | -.367 | -.052 | .610 | .628 | .730 | .077 | .608 | -.256 | .201 | -.569 | .600 | .638 |
| pH |  | 1 | .584 | -.450 | -.459 | .065 | .559 | .315 | .389 | -.412 | .068 | -.461 | -.226 |
| DO |  |  | 1 | -.044 | -.052 | -.155 | .757 | .006 | .699 | .420 | .471 | -.016 | -.362 |
| EC |  |  |  | 1 | 1.000^**^ | .033 | .405 | -.049 | .336 | .436 | -.382 | .780 | .631 |
| TDS |  |  |  |  | 1 | .051 | .395 | -.035 | .319 | .436 | -.397 | .789 | .644 |
| Phosphate |  |  |  |  |  | 1 | -.153 | .966^**^ | -.599 | -.447 | -.589 | -.015 | .398 |
| Nitrate |  |  |  |  |  |  | 1 | .018 | .881^*^ | .303 | -.027 | .330 | .182 |
| TP |  |  |  |  |  |  |  | 1 | -.453 | -.536 | -.539 | -.134 | .321 |
| TKN |  |  |  |  |  |  |  |  | 1 | .501 | .273 | .301 | -.044 |
| Taxa_S |  |  |  |  |  |  |  |  |  | 1 | .327 | .661 | .007 |
| Individuals |  |  |  |  |  |  |  |  |  |  | 1 | -.480 | -.934^**^ |
| Shannon_H |  |  |  |  |  |  |  |  |  |  |  | 1 | .754 |
| Evenness |  |  |  |  |  |  |  |  |  |  |  |  | 1 |

**. Correlation is significant at the 0.01 level (2-tailed); *. Correlation is significant at the 0.05 level (2-tailed).

**c. The correlation between bioindices and physicochemical parameters of the six studied wetlands during wet seasons**

|  | Temperature | pH | DO | EC | TDS | Phosphate | Nitrate | TP | TKN | Taxa_S | Individuals | Shannon_H | Evenness |
| --- | --- | --- | --- | --- | --- | --- | --- | --- | --- | --- | --- | --- | --- |
| Temperature | 1 | -.711 | -.156 | .598 | .594 | -.808 | -.122 | -.683 | -.786 | .432 | .690 | -.021 | -.457 |
| pH |  | 1 | .166 | -.634 | -.598 | .681 | -.039 | .575 | .338 | -.612 | -.669 | .200 | .737 |
| DO |  |  | 1 | -.733 | -.739 | .408 | -.371 | .533 | .543 | -.208 | -.330 | -.632 | -.487 |
| EC |  |  |  | 1 | .998^**^ | -.581 | .426 | -.645 | -.701 | .727 | .754 | .596 | .046 |
| TDS |  |  |  |  | 1 | -.561 | .444 | -.628 | -.716 | .713 | .740 | .626 | .086 |
| Phosphate |  |  |  |  |  | 1 | .414 | .960^**^ | .787 | -.404 | -.772 | -.008 | .364 |
| Nitrate |  |  |  |  |  |  | 1 | .395 | .120 | .119 | -.190 | .407 | .314 |
| TP |  |  |  |  |  |  |  | 1 | .817^*^ | -.506 | -.840^*^ | -.234 | .165 |
| TKN |  |  |  |  |  |  |  |  | 1 | -.424 | -.733 | -.487 | -.132 |
| Taxa_S |  |  |  |  |  |  |  |  |  | 1 | .879^*^ | .478 | -.200 |
| Individuals |  |  |  |  |  |  |  |  |  |  | 1 | .392 | -.241 |
| Shannon_H |  |  |  |  |  |  |  |  |  |  |  | 1 | .754 |
| Evenness |  |  |  |  |  |  |  |  |  |  |  |  | 1 |

**. Correlation is significant at the 0.01 level (2-tailed); *. Correlation is significant at the 0.05 level (2-tailed).
