# Supplementary material for: Composition and dynamics of macroinvertebrates community in relation to physicochemical parameters of hydrogeologically connected wetlands in Abbay River basin, Ethiopia
Source: PLoS One. 2024 Dec 9;19(12):e0314969. doi: 10.1371/journal.pone.0314969 (PMC11627383; doi:10.1371/journal.pone.0314969)
Supplement: S2 Table — (DOCX) [file pone.0314969.s002.docx]

**SI_Table 2. Macroinvertebrates collected from the study wetlands across the study seasons**

| **Phylum** | **Class** | **Order** | **Family** | **Geray** | | **Gudera** | | **Zindib** | | **Kbahir** | | **Infranz** | | **Wonjeta** | | **Total** | **RA** |
| --- | --- | --- | --- | --- | --- | --- | --- | --- | --- | --- | --- | --- | --- | --- | --- | --- | --- |
|  |  |  |  | **Wet** | **Dry** | **Wet** | **Dry** | **Wet** | **Dry** | **Wet** | **Dry** | **Wet** | **Dry** | **Wet** | **Dry** |  |  |
| **Arthropoda** | **Insecta** | **Coleoptera** | Hydrophilidae | 0 | 0 | 0 | 2 | 0 | 13 | 0 | 11 | 7 | 0 | 17 | 4 | **54** | **4.46** |
|  |  |  | Dytiscidae | 16 | 8 | 2 | 20 | 6 | 13 | 5 | 15 | 9 | 13 | 7 | 3 | **117** | **9.66** |
|  |  |  | Gyrinidae | 0 | 0 | 0 | 0 | 0 | 1 | 0 | 1 | 0 | 1 | 0 | 1 | **4** | **0.33** |
|  |  |  | Noteridae | 0 | 0 | 0 | 0 | 0 | 0 | 0 | 1 | 0 | 4 | 0 | 4 | **9** | **0.74** |
|  |  |  | Hydraenidae | 1 | 0 | 0 | 0 | 0 | 0 | 0 | 0 | 0 | 0 | 0 | 0 | **1** | **0.08** |
|  |  |  | Elmidae | 0 | 0 | 0 | 0 | 0 | 0 | 0 | 2 | 0 | 0 | 0 | 0 | **2** | **0.17** |
|  |  | **Diptera** | Chironomidae | 0 | 5 | 0 | 33 | 0 | 1 | 23 | 13 | 1 | 1 | 7 | 25 | **109** | **9.00** |
|  |  |  | Culicidae | 0 | 0 | 0 | 1 | 0 | 0 | 0 | 0 | 0 | 0 | 0 | 0 | **1** | **0.08** |
|  |  |  | Simuliidae | 0 | 1 | 0 | 0 | 0 | 0 | 0 | 0 | 0 | 0 | 0 | 0 | **1** | **0.08** |
|  |  |  | Chaoboridae | 0 | 0 | 1 | 0 | 0 | 0 | 0 | 0 | 0 | 0 | 0 | 0 | **1** | **0.08** |
|  |  | **Ephemeroptera** | Ephemerelidae | 0 | 0 | 0 | 0 | 0 | 0 | 0 | 5 | 0 | 1 | 0 | 0 | **6** | **0.50** |
|  |  |  | Teloganodidae | 0 | 0 | 0 | 0 | 0 | 0 | 0 | 0 | 0 | 5 | 0 | 0 | **5** | **0.41** |
|  |  |  | Baetidae | 0 | 0 | 1 | 0 | 0 | 0 | 0 | 0 | 0 | 0 | 2 | 0 | **3** | **0.25** |
|  |  | **Hemiptera** | Corixidae | 0 | 11 | 1 | 75 | 0 | 42 | 4 | 5 | 4 | 1 | 1 | 17 | **161** | **13.29** |
|  |  |  | Gerridae | 2 | 3 | 0 | 1 | 0 | 5 | 0 | 0 | 3 | 1 | 0 | 0 | **15** | **1.24** |
|  |  |  | Notonectidae | 24 | 26 | 43 | 29 | 0 | 28 | 20 | 45 | 4 | 9 | 12 | 5 | **245** | **20.23** |
|  |  |  | Nepidae | 1 | 0 | 1 | 0 | 0 | 2 | 0 | 2 | 0 | 0 | 0 | 0 | **6** | **0.50** |
|  |  |  | Naucoridae | 0 | 2 | 0 | 0 | 0 | 0 | 0 | 3 | 0 | 5 | 18 | 1 | **29** | **2.39** |
|  |  |  | Veliidae | 0 | 2 | 0 | 1 | 0 | 0 | 0 | 0 | 0 | 10 | 0 | 5 | **18** | **1.49** |
|  |  |  | Mesoveliidae | 0 | 0 | 0 | 1 | 0 | 0 | 0 | 0 | 0 | 0 | 0 | 0 | **1** | **0.08** |
|  |  | **Lepidoptera** | Crambidae | 0 | 1 | 0 | 0 | 0 | 0 | 0 | 0 | 0 | 0 | 0 | 0 | **1** | **0.08** |
|  |  | **Odonata** | Aeshnidae | 1 | 4 | 0 | 1 | 0 | 7 | 0 | 5 | 4 | 5 | 2 | 1 | **30** | **2.48** |
|  |  |  | Gomphidae | 0 | 6 | 3 | 10 | 10 | 5 | 0 | 12 | 15 | 25 | 0 | 16 | **102** | **8.42** |
|  |  |  | Libellulidae | 0 | 5 | 0 | 1 | 0 | 1 | 0 | 4 | 0 | 13 | 0 | 0 | **24** | **1.98** |
|  |  |  | Corduliidae | 0 | 0 | 0 | 0 | 0 | 0 | 0 | 2 | 0 | 12 | 0 | 2 | **16** | **1.32** |
|  |  |  | Calopterygidae | 0 | 0 | 0 | 0 | 0 | 0 | 0 | 2 | 0 | 2 | 0 | 0 | **4** | **0.33** |
|  |  |  | Coenagrionidae | 0 | 22 | 0 | 1 | 2 | 15 | 0 | 7 | 35 | 15 | 3 | 3 | **103** | **8.51** |
|  |  |  | Lestidae | 6 | 0 | 2 | 2 | 2 | 0 | 0 | 2 | 0 | 2 | 0 | 0 | **16** | **1.32** |
|  |  | **Plecoptera** | Leuctridae | 0 | 0 | 0 | 0 | 0 | 0 | 0 | 5 | 0 | 0 | 0 | 0 | **5** | **0.41** |
|  |  | **Trichoptera** | Psychomyiidae | 0 | 0 | 0 | 0 | 0 | 0 | 0 | 0 | 0 | 1 | 0 | 0 | **1** | **0.08** |
|  |  |  | Hydropsychidae | 0 | 0 | 0 | 0 | 0 | 0 | 0 | 0 | 1 | 0 | 0 | 0 | **1** | **0.08** |
|  |  |  | Hydroptilidae | 0 | 0 | 0 | 0 | 0 | 0 | 2 | 0 | 0 | 0 | 0 | 0 | **2** | **0.17** |
|  | **Crusteca** | **Decapoda** | Amphipoda | 0 | 0 | 0 | 0 | 0 | 0 | 2 | 0 | 0 | 0 | 0 | 0 | **2** | **0.17** |
|  |  |  | Potamonautidae | 0 | 0 | 0 | 0 | 7 | 2 | 0 | 0 | 1 | 0 | 0 | 0 | **10** | **0.83** |
|  | **Arachnida** | **Araneae** | Pisauridae | 0 | 0 | 0 | 0 | 0 | 0 | 0 | 0 | 0 | 0 | 0 | 1 | **1** | **0.08** |
| **Annelid** | **Oligochaete** | **Opisthopora** | Earthworm | 0 | 0 | 0 | 0 | 0 | 0 | 0 | 0 | 0 | 1 | 1 | 0 | **2** | **0.17** |
|  | **Hirudinea** | **Hirudinida** | Hirudinea | 1 | 0 | 0 | 3 | 0 | 0 | 0 | 0 | 0 | 0 | 0 | 1 | **5** | **0.41** |
| **Mollusca** | **Gastropoda** | **Basommatophora** | Planorbidae | 0 | 1 | 0 | 2 | 0 | 0 | 0 | 4 | 1 | 2 | 0 | 10 | **20** | **1.65** |
|  |  | **Hygrophila** | Lymnaeidae | 1 | 1 | 1 | 2 | 2 | 3 | 0 | 0 | 0 | 1 | 0 | 8 | **19** | **1.57** |
|  |  |  | Physidae | 0 | 0 | 0 | 0 | 1 | 1 | 3 | 0 | 6 | 0 | 1 | 1 | **13** | **1.07** |
|  |  | **Neotaenioglossa** | Thiaridae | 0 | 0 | 0 | 0 | 0 | 0 | 0 | 0 | 0 | 1 | 0 | 0 | **1** | **0.08** |
|  | **Bivalvia** | **Sphaeriida** | Sphaeriidae | 0 | 0 | 0 | 34 | 0 | 0 | 0 | 0 | 0 | 0 | 0 | 3 | **37** | **3.06** |
|  |  | **Venerida** | Corbiculidae | 0 | 0 | 0 | 6 | 0 | 0 | 0 | 0 | 0 | 0 | 0 | 0 | **6** | **0.50** |
| **Nematode** | **Chromadorea** | **Ascaridida** | Ascarididae | 0 | 0 | 0 | 2 | 0 | 0 | 0 | 0 | 0 | 0 | 0 | 0 | **2** | **0.17** |
| **Total Individuals per season** | | | | **53** | **98** | **55** | **227** | **30** | **139** | **59** | **146** | **91** | **131** | **71** | **111** | **1211** | |
| **Total individuals across wetlands** | | | | **151** | | **282** | | **169** | | **205** | | **222** | | **182** | |  |  |
